# The Concentration of Non-structural Carbohydrates, N, and P in *Quercus variabilis* Does Not Decline Toward Its Northernmost Distribution Range Along a 1500 km Transect in China

**DOI:** 10.3389/fpls.2018.01444

**Published:** 2018-10-17

**Authors:** Jian-Feng Liu, Yun-Peng Deng, Xiao-Fei Wang, Yan-Yan Ni, Qi Wang, Wen-Fa Xiao, Jing-Pin Lei, Ze-Ping Jiang, Mai-He Li

**Affiliations:** ^1^State Key Laboratory of Tree Genetics and Breeding, Key Laboratory of Tree Breeding and Cultivation of State Forestry Administration, Research Institute of Forestry, Chinese Academy of Forestry, Beijing, China; ^2^Research Institute of Forest, Ecology and Environment Protection, Chinese Academy of Forestry, Beijing, China; ^3^Guangdong Institute of Eco-environmental Science and Technology, Guangzhou, China; ^4^Swiss Federal Research Institute WSL, Birmensdorf, Switzerland; ^5^School of Geographical Sciences, Northeast Normal University, Changchun, China

**Keywords:** *Quercus variabilis*, latitudinal distribution range, ontogeny, non-structural carbohydrate, nutrient

## Abstract

Understanding the mechanisms that determine plant distribution range is crucial for predicting climate-driven range shifts. Compared to altitudinal gradients, less attention has been paid to the mechanisms that determine latitudinal range limit. To test whether intrinsic resource limitation contributes to latitudinal range limits of woody species, we investigated the latitudinal variation in non-structural carbohydrates (NSC; i.e., total soluble sugar plus starch) and nutrients (nitrogen and phosphorus) in mature and juvenile Chinese cork oak (*Quercus variabilis* Blume) along a 1500 km north-south transect in China. During the growing season and dormant season, leaves, branches, and fine roots were collected from both mature and juvenile oaks in seven sites along the transect. Tissue concentration of NSCs, N, and P did not decrease with increasing latitude irrespective of sampling season and ontogenetic stage. Furthermore, higher levels of NSCs and N in tissues of juveniles relative to mature trees were found during the dormant season. Partial correlation analysis also revealed that during the dormant season, soluble sugar, NSC, the ratio of soluble sugar to starch, and tissue nitrogen concentration were correlated positively with latitude but negatively with precipitation and mean temperature of dormant season. Our results suggest that carbon or nutrient availability may not be the driving factors of the latitudinal range limit of the studied species. Further studies should be carried out at the community or ecosystem level with multiple species to additionally test the roles of factors such as regeneration, competition, and disturbance in determining a species’ northern distribution limit.

## Introduction

In the context of climate change, altitudinal and latitudinal gradients can be used as natural laboratories to deduce species’ responses to global warming (Körner, 2007; De Frenne et al., 2013). Nowadays, northward and upward shift of species distribution has been observed in a wide range of studies (Kelly and Goulden, 2008; Chen et al., 2011; Boisvert-Marsh et al., 2014; Desprez et al., 2014; Rinas et al., 2017; Sittaro et al., 2017), but the underlying physiological mechanisms are under debate. Therefore, we may use geographical gradients to explore the mechanisms for species’ distribution under current conditions, and thus to understand and forecast the responses of species’ distribution to predicted climate change.

Compared to a few eco-physiological evidences for the formation of a species’ northernmost distribution, altitudinal tree-line formation has attracted lots of studies during the last several decades, and several important hypotheses have been proposed (Li and Krauchi, 2005). Notably, two mutually exclusive hypotheses: the Carbon Limitation Hypothesis (CLH) (Stevens and Fox, 1991) and the Growth Limitation Hypothesis (GLH) (Körner, 1998; Hoch and Körner, 2003), have been extensively tested, as they both have the potential to be applied worldwide (Körner, 1998) due to intrinsic response or adaptation strategies of plants to a variety of environmental factors at the alpine tree-line. Non-structural carbohydrates (NSC, sum of starch and total soluble sugars) and nutrients (nitrogen or phosphorus) of plant tissues were generally used to evaluate the carbon or nutrient status along altitudinal gradients to test the hypotheses mentioned above (Hoch and Körner, 2003, 2012; Li et al., 2008a; Sullivan et al., 2014; Fajardo and Piper, 2017). NSC, formed during photosynthesis, could act as substrate for respiration to provide the energy needed for growth and maintenance processes (Körner, 2003); and the level of its two main components, soluble sugars and starch, could reflect the balance between carbon gain and carbon utilization and loss within a plant, representing a tree’s capital for growth and acting as a buffer during insufficient source activities due to environmental stress (Li et al., 2002; O’Brien et al., 2014). Meanwhile, nitrogen and phosphorus are the two most limiting elements to terrestrial vegetation (Reich and Oleksyn, 2004), which are not only directly related to carbon assimilation and allocation but also indirectly to stress tolerance or resistance (DeHayes et al., 1989; Villagra et al., 2013; Yan et al., 2016). However, at a single species level, whether the factors in determining the latitudinal distribution range are also associated with carbon and nutrient status still remain unclear.

Apart from the vital roles of carbon and nutrients to support plant survival and growth, ontogenetic variations in responses to environment variability or stress have gained more attention (Niinemets, 2010; le Roux et al., 2013; Klockmann et al., 2017). To date, numerous studies have already reported ontogenetic variations in carbon assimilation and allocation (Portsmuth et al., 2005; Steppe et al., 2011), resource use strategies (Gedroc et al., 1996; Rivas-Ubach et al., 2012), and stress tolerances (Cavender-Bares and Bazzaz, 2000; Niinemets, 2010). Compared to adult trees, however, less attention has been paid to early life stages (e.g., seedlings or saplings), which are more sensitive to environmental changes or stresses (Niinemets, 2010). Although adult trees may persist over hundreds of years, if seedlings or saplings fail to keep pace with the rate of rising temperature or climate change (Zhu et al., 2012; Bell et al., 2014; Máliš et al., 2016), in the long-term the distribution range of a species will shrink. Hence, to predict the responses of tree species to climate change, disentangling ontogenetic variations along environmental gradients is indispensable for a more profound understanding of adaptation strategies among different life stages, as well as for a better understanding of the mechanisms in determining the latitudinal range.

The Chinese cork oak (*Quercus variabilis* Blume) is one of the most widely distributed tree species in eastern Asia, stretching from approximately 24° to 40°N and 97° to 140°E (Chen et al., 2012). The different regions where the species is present differ greatly in terms of climatic and edaphic conditions, providing an ideal situation for studying the spatial patterns of carbon or nutrient allocation within a single widespread species. In the present study, leaves, branches, and fine roots in healthy juvenile and mature trees in seven sites along a designed 1500 km north-south transect in China, were collected and analyzed. Our aims were to answer the questions of (1) how the carbon or nutrient status vary along the south-north transect; and (2) whether there is a consistent difference in NSCs and nutrients between juvenile and mature trees across latitudes. Our results will contribute toward understanding and predicting the impacts of climate change on the range dynamics of woody plants along latitudinal gradients.

## Materials and Methods

### Sampling Protocol

**FIGURE 1:**
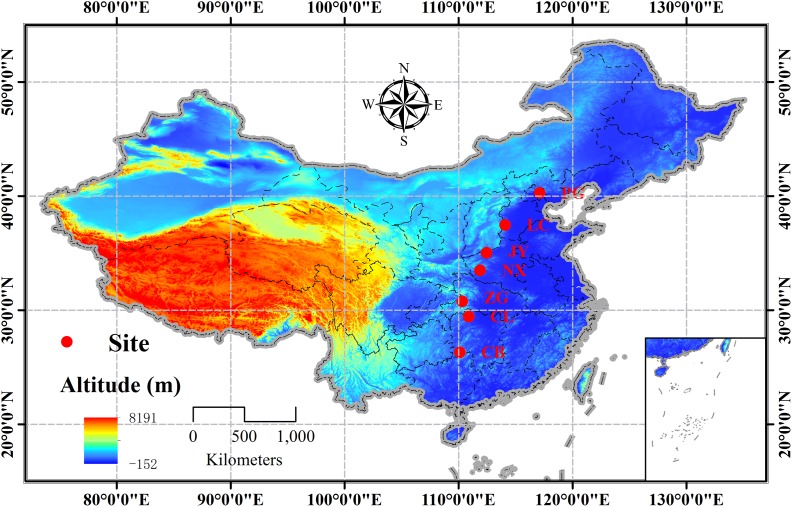
Distribution of the sampling sites of *Quercus variabilis* in China (PG: Pinggu, Beijing; LC: Lincheng, Hebei; JY: Jiyuan, Henan; NX: Neixiang, Henan; ZG: Zigui, Hubei; CL: Cili, Hunan; and CB: Chengbu, Hunan).

A south-north transect covering 14 latitudinal degrees (∼26°–40°N) (c.1500 km) was setup in the natural distribution range of *Q. variabilis* in China, and seven sites were designed along the transect with a latitudinal interval of ∼2° (**Figure 1** and **Supplementary Table S1**). Using the geographical location-based method by Gong and Jian (1983), we calculated the phenological date for each site (**Supplementary Table S1**), so that we collected samples at the same phenological stage across the seven sites to avoid effects of plant phenological variation or bias on carbon or nutrient status (Bansal and Germino, 2009; Bazot et al., 2013). The sampling tasks were conducted on two age classes (juvenile and mature) of natural stands during the mid-growing season (August, 2014) and the dormant season (Dec, 2014). At each site, three 50 m × 50 m temporal plots (*n* = 3), with a minimum distance of 10 km from each other, were set up. Within each plot, three to four canopy trees of the same age class, without browsing and other damages, were selected. The sampling processes were identical for the two sampling seasons. From each sample tree, three to four upper and outer-most sun-exposed branches were cut to collect the leaves (only for growing season) and branches (1-2a). Fine roots (<5 mm in diameter) attached to coarse roots of each sample tree were manually excavated using a mini-spade and carefully collected. All samples were stored in a cool box until they were taken to the laboratory. The same tissue from individuals in the same age class collected from the same site was pooled as one mixed sample. All samples were heated in a microwave oven at 600 W for 60 s, and then dried at 65°C for 72 h and ground to pass a 0.20 mm sieve for further analyses.

### Biochemical Analysis

#### Total Soluble Sugars and Starch

The powdered material (∼0.10 g) was put into a 10 ml centrifuge tube, and 5 ml of 80% ethanol was added. The mixture was incubated at 80°C in a water bath shaker for 30 min, and then centrifuged at 4000 rpm for 5 min. The pellets were extracted two more times with 80% ethanol. Supernatants were retained, combined, and stored at -20°C for soluble sugar determinations. The ethanol-insoluble pellet was used for starch extraction. Glucose was used as a standard. Soluble sugars were determined using the anthrone method (Seifter et al., 1950). The starch concentration was measured spectrophotometrically at 620 nm using anthrone reagent, and was calculated by multiplying the glucose concentrations by the conversion factor of 0.9 (Osaki et al., 1991). The concentration of soluble sugars and starch was described on a dry matter basis (mg g^-1^ DW).

#### Total Nitrogen and Phosphorus

For the determination of tissues’ nitrogen (N) and phosphorus (P) concentrations (mg g^-1^ DW), finely ground material (∼50 mg) was first digested with H_2_SO_4_ and then H_2_O_2_ for further analysis. The nitrogen concentration was then measured using the Kjeldahl method (Kjeltec 2200, FOSS, Sweden), while the phosphorus concentration was determined with the molybdenum blue spectrophotometric procedure (6505 UV spectrophotometer, United Kingdom) (Page, 1982).

#### Environmental Data

The monthly climatic data (2014) were interpolated with the kriging method (Matheron, 1963) from 675 national weather stations around China (download from^1^) using GIS software (ArcGIS v10.0, Esri, United States). The soil data was obtained from the gridded Global Soil dataset (30 arc-second resolution) which was developed by Shangguan et al. (2014)^2^. The special values of each sampled plot were then extracted according to the geolocation information (latitude and longitude) of the plot. The climatic variables, monthly mean temperature (°C) and monthly mean precipitation (mm), were divided into two parts as one for the growing season (April to September; represented as gT mean and gPPT) and another for the dormant season (October to December; denoted as dT mean and dPPT). The soil variables, on the other hand, included total nitrogen (SN, %) and total phosphorus (SP, %) for each plot.

### Statistical Analyses

All statistical analyses were conducted using R statistical software (RStudio version 1.0.143^3^). Shapiro-Wilk and Bartlett’s tests were used to test for normality and homogeneity of variances, respectively, and it was found that all data met the assumption for further variance analysis. We utilized a linear mixed effects model with tree age (juvenile and mature trees), sampling site (latitudinal gradient), and season (growing season: Aug; and dormant season: December) as fixed effects and individuals as a random effect (R package ‘lme4’). The response variables included soluble sugar, starch, NSC, nitrogen, and phosphorus in the current-year leaves, branches, and fine roots. The mean and standard error were given if necessary. For both the sampling seasons, a partial pairwise correlation analysis was performed to explore the correlations between the response variables with the environmental factors (e.g., climate or soil), where bio-factors (tree age and tree tissue) were treated as covariant (R package ‘psych’). The contributions of all the environmental and biological factors to variations of responsible variables were performed by the redundancy analysis (RDA). The variation partitioning was achieved by means of partial redundancy analysis (pRDA) to extract the pure effect of environment factors and the pure effect of bio-factors (R package ‘vegan’).

## Results

### Non-structural Carbohydrate and Its Components

**FIGURE 2:**
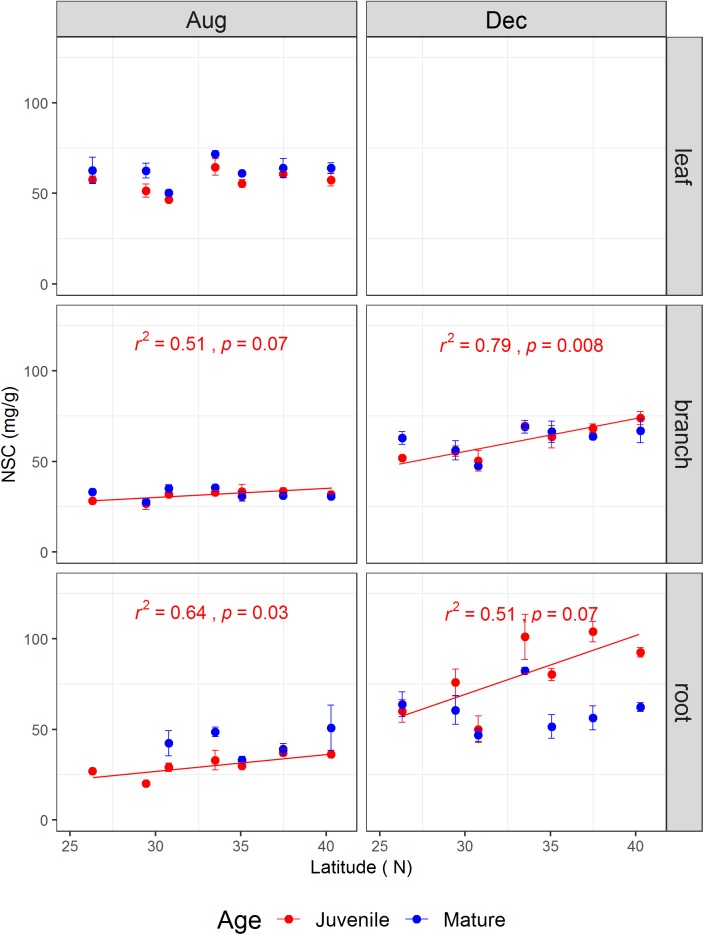
The non-structural carbohydrate concentrations (mg/g) (mean ± se, *n* = 3) across life stages (juveniles vs. mature), tissues (leaf, branch, and fine root), and sampling seasons (growing season: Aug, 2014; and dormant season: Dec, 2014) along the latitudinal gradient. For each subplot, blue color denotes mature individuals and red denotes juveniles; fitted curves, determinant coefficients, and *p*-values of simple linear regression are given.

The tree age significantly affected leaf soluble sugars and leaf NSC concentration, as well as branch starch, but did not impact root NSCs (**Table 1**). The sampling season and the interaction between sampling season and tree age significantly influenced the NSCs in the branches and fine roots (**Table 1**). For instance, root NSC in both juveniles and mature trees was significantly higher in December than in August (*p* < 0.001) (**Supplementary Figure S1**). However, during the growing season, root NSC in juveniles (30.34 ± 1.46 mg g^-1^) was significantly lower than that in mature trees (42.79 ± 3.06 mg g^-1^, *p* < 0.05), whereas it showed an opposite result (80.61 ± 4.79 mg g^-1^ for juveniles and 60.52 ± 2.94 mg g^-1^ for mature trees, *p* < 0.001) during the dormant season. On the other hand, the NSCs including the ratio of soluble sugars to starch varied strongly with latitude and tissue type (**Figure 2** and **Supplementary Figures S2–S4**). The results from simple linear models indicated that the NSC of various tissues for both life stages did not show any decreasing trend with increasing latitude (**Figure 2**). Inversely, the NSC in the branches and roots of juveniles significantly or marginally significantly increased with the increasing latitude for both sampling seasons (**Figure 2**).

**Table 1:** Effects of age (*juvenile* and *mature*), latitude, sampled season (growing season: Aug, 2014; and dormant season: Dec, 2014) and their interactions on NSCs, nutrients over tissues revealed by linear mixed effects models with individuals as random factors.

Source of variation		Soluble sugar		Starch		NSC		SS		Nitrogen		Phosphorus		N:P	
	*df*	*F*	*P*	*F*	*P*	*F*	*P*	*F*	*P*	*F*	*P*	*F*	*P*	*F*	*P*
**Leaf**								
Age	1	9.705	**0.004**	1.526	0.227	10.683	**0.003**	7.415	**0.011**	1.449	0.232	10.571	**0.003**	7.447	**0.011**
Latitude	6	4.747	**0.002**	5.247	**0.001**	5.973	**0.000**	3.241	**0.015**	5.524	**0.001**	3.228	**0.015**	6.666	**0.000**
Age × latitude	6	0.714	0.642	1.873	0.121	0.281	0.941	1.136	0.367	4.135	**0.005**	2.174	0.076	2.820	**0.028**
**Branch**								
Age	1	3.042	0.087	4.820	**0.032**	0.094	0.761	2.161	0.147	0.094	0.761	118.33	**0.000**	35.730	**0.000**
Latitude	6	20.027	**0.000**	18.205	**0.000**	7.386	**0.000**	24.459	**0.000**	6.468	**0.000**	14.958	**0.000**	16.909	**0.000**
Season	1	455.16	**0.000**	175.37	**0.000**	607.45	**0.000**	2.022	0.161	4.920	**0.031**	98.364	**0.000**	15.422	**0.000**
Age × latitude	6	1.111	0.368	2.507	**0.032**	1.464	0.207	1.325	0.261	1.255	0.293	12.790	**0.000**	5.282	**0.000**
Age × season	1	3.996	**0.050**	15.175	**0.000**	0.136	0.714	9.841	**0.003**	3.528	0.066	90.777	**0.010**	16.029	**0.000**
Latitude × season	6	6.261	**0.000**	10.860	**0.000**	5.602	**0.000**	10.797	**0.000**	1.528	0.186	10.381	**0.000**	2.563	**0.029**
Age × latitude × season	6	0.549	0.769	0.768	0.598	0.567	0.755	0.166	0.985	0.882	0.514	11.385	**0.000**	3.907	**0.002**
**Root**								
Age	1	0.015	0.903	1.378	0.246	1.093	0.301	3.741	0.059	4.051	**0.049**	47.490	**0.000**	93.730	**0.000**
Latitude	6	10.473	**0.000**	2.674	**0.025**	8.357	**0.000**	3.331	**0.008**	6.754	**0.000**	2.342	**0.044**	13.648	**0.000**
Season	1	347.83	**0.000**	34.088	**0.000**	263.20	**0.000**	6.886	**0.011**	16.685	**0.000**	28.173	**0.000**	0.183	0.670
Age × latitude	6	1.374	0.243	4.389	**0.001**	2.646	**0.026**	4.572	**0.001**	0.534	0.780	2.990	**0.013**	4.253	**0.001**
Age × season	1	69.450	**0.000**	5.537	**0.022**	49.532	**0.000**	7.979	**0.007**	2.950	0.091	23.975	**0.000**	34.849	**0.000**
Latitude × season	6	5.011	**0.000**	4.091	**0.002**	5.023	**0.000**	3.310	**0.008**	1.297	0.274	2.722	**0.022**	3.571	**0.005**
Age × latitude × season	6	1.489	0.219	2.309	0.070	1.271	0.293	1.556	0.200	1.026	0.418	2.057	**0.073**	1.500	0.196


*Significance levels of less than 0.05 are identified in bold.*

### Nitrogen, Phosphorus Concentrations and Their Ratios

**FIGURE 3:**
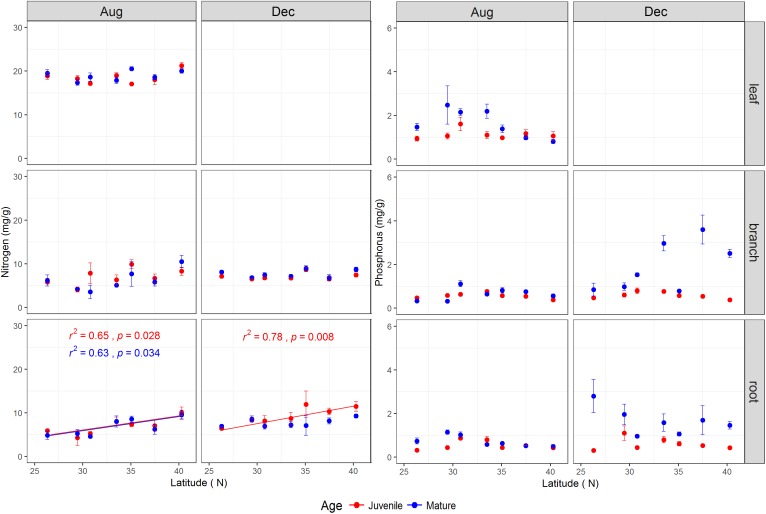
The total nitrogen and phosphorus concentrations (mg/g) (mean ± se, *n* = 3) across life stages (juveniles vs. mature), tissues (leaf, branch, and fine root), and sampling seasons (growing season: Aug, 2014; and dormant season: Dec, 2014) along the latitudinal gradient. For each subplot, blue color denotes mature individuals and red denotes juveniles; fitted curves, determinant coefficients, and *p*-values of simple linear regression are given.

The tree age had significant impacts on N, P, and N:P ratio of the studied tissues with exception of leaf N and branch N (**Table 1** and **Supplementary Figure S5**). Mature trees generally had higher P concentration across tissues than juveniles, especially during the dormant season (**Supplementary Figure S5**). During the growing season, the N, P, and N:P ratio in the leaves of mature trees were 18.93 ± 0.32 mg g^-1^, 1.63 ± 0.18 mg g^-1^, and 14.27 ± 1.48, respectively, whereas, the corresponding values for juveniles were 18.54 ± 0.36 mg g^-1^, 1.13 ± 0.07 mg g^-1^, and 17.71 ± 1.08, respectively (**Supplementary Figure S5**). The P and N:P ratio in various tissues were interactively affected by tree age and latitude (**Table 1**), and N:P ratio in the leaves and roots of mature trees during the growing season increased significantly with latitude (**Supplementary Figure S6**). The tissues’ N varied significantly with latitude (**Table 1** and **Figure 3**), while root N of both life stages increased with increasing latitude for both sampling seasons with the exception of mature trees’ roots in the dormant season (**Figure 3**).

### Correlation Between Non-carbohydrates, Nutrients, and Environmental Factors

**FIGURE 4:**
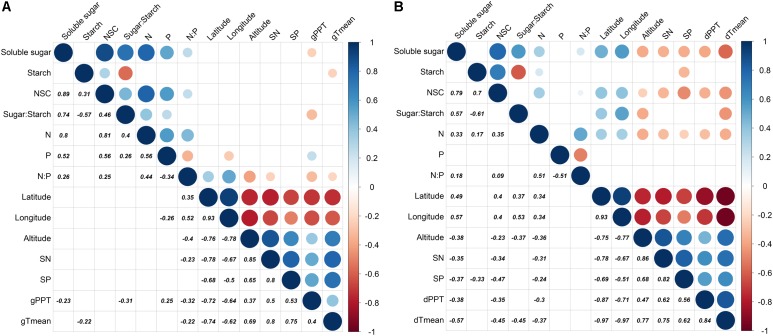
Partial correlation analysis for dependent variables (soluble sugar, starch, NSC, ratio of sugar to starch, N, P, and ratio of N to P) and environmental factors (latitude, longitude, elevation, soil N, soil P, precipitation, and monthly mean temperature) where tree age and tree tissue were regarded as control factors [**(A)**: growing season; **(B)**: dormant season]. Significantly positive or negative correlations (*p* < 0.05) are displayed in blue and in red color, respectively; and color intensity and circle size are proportional to the correlation coefficients.

**FIGURE 5:**
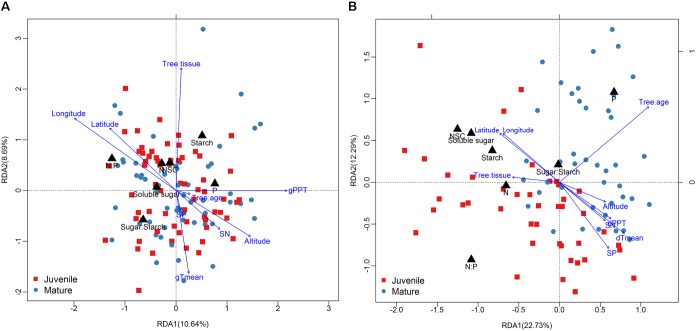
Results of redundancy analysis [**(A)**: growing season; **(B)**: dormant season] showing samples (mature tree: dark blue points; juvenile tree: red square), responsive variables (black triangle), and affecting factors (blue arrow).

The season transition strongly affected the intensity of correlations between dependent variables with environmental variables (**Figures 4, 5**). During the growing season, tissues’ NSC and N have no significant correlations with the environmental factors, e.g., geographical, soil, or climatic variables (**Figure 4A**). The ratios of soluble sugar to starch were negatively correlated with precipitation of growing season. The ratios of N to P were positively correlated with latitude and longitude but negatively correlated with elevation, soil N, precipitation, and mean temperature of growing season (**Figure 4A**). The partial RDA revealed that only a total of 26.2% variations of dependent variables were jointly explained by environmental factors (17.5%) and bio-factors (8.3%) (**Supplementary Figure S7A**). Nevertheless, during the dormant season, soluble sugar, NSC, the ratio of soluble sugar to starch and tissue nitrogen were correlated positively with latitude and longitude but negatively with elevation, soil N, soil P, precipitation, and mean temperature of dormant season (**Figure 4B**). A total of 48.2% variations of dependent variables were jointly explained by environmental (27.5%) and bio-factors (20.6%) (**Supplementary Figure S7B**).

## Discussion

The present study showed that NSCs (total soluble sugars, starch, and NSC) in woody tissues (branches and fine roots) of both life stages had higher concentrations in the dormant season than in the growing season along the latitudinal gradient (**Supplementary Figure S1**). Our results were consistent with Martínez-Vilalta et al. (2016) who complied data from 121 studies including 177 species under natural conditions and found that NSCs varied seasonally, with a general increase during winter months. Compared to evergreen species, deciduous trees generally stored more NSC in the tissues (e.g., stem or root) to withstand low temperature in the coming winter and to support bud-break and shoot growth in the early spring (Klein et al., 2016). On the other hand, higher level of NSC in reserved tissues during the dormant season may attribute to lower level of growth and maintenance respiration due to low temperature in the winter, with more starch broken down into soluble sugars to promote cold tolerance by adjusting the intracellular osmotic concentration (Morin et al., 2007), as shown by an increase in the sugar-starch ratios in juvenile roots in our study (**Supplementary Figure S4**). Juveniles’ roots had lower NSC concentration in the growing season but higher NSC concentration in the dormant season than that of mature individuals, especially for north populations (**Figure 2**), indicating that juveniles are more sensitive to seasonal transition than mature trees. This further suggests that juveniles may use a strategy to invest more NSC into fast growth (thus leading to lower level of NSC in juveniles than in mature trees during the growing season) to get a competitive advantage with relatively larger body size in a community. During the dormant season, juveniles generally had a higher level of NSC to cope with low temperature because of their relatively younger tissue and smaller body size (i.e., smaller carbon pool size) (Bansal and Germino, 2009, 2010). On the other hand, Li et al. (2008b) proposed that trees growing at the elevational or the latitudinal climate limit rely not only on the total NSC concentration but also require a sufficiently high sugar-starch ratio to overwinter successfully. Our results support this view, as both life stages maintained comparable high ratio of soluble sugars to starch in the dormant season (**Supplementary Figure S1**).

Although there exists considerable disparities between altitudinal and latitudinal gradients (Jump et al., 2009), both gradients are mainly dominated by temperature. In this regard, the altitudinal tree-line hypotheses (e.g., GLH and CLH) present important reference values to deduce the driven mechanism for latitudinal range. In the present study, we found that tissue NSC did not exhibit decreasing trends for both life stages along the latitudinal gradient. Nevertheless, NSC in the woody tissues of juveniles increased significantly with increasing latitude during both the sampling seasons, suggesting that carbon limitation cannot act as a determinant driver to north latitudinal range of the species studied. This result fails to support the CLH along latitudinal gradients. Li et al. (2016) also observed weekly increasing latitudinal trends of leaf NSCs from tropical to cold temperate forests at the levels of species and plant functional groups. Alternatively, other factors, such as recruitment limitation, rather than NSC at the north edges may contribute to the oak’s north-latitudinal limit formation. Our previous study found that the relative densities of seedlings of the species studied were significantly lower in the northern edge than in the core populations (Gao et al., 2017).

Apart from the role of carbon status in determining the range limit, nutrient shortage is another important factor to elucidate the issue which not only limited carbon assimilation but also limited tissue development (McNown and Sullivan, 2013; Sullivan et al., 2014). Ontogenetic variations in tissue P but not in tissue N were found in this study (**Supplementary Figure S5**), where mature trees had higher tissue P concentration than juveniles, especially during the dormant season, consequently leading to relatively lower N:P ratio occurring in mature trees. This result is inconsistent with Noh et al. (2007) who found that N and P concentrations in tissues of *Q. acutissima* significantly decreased with tree age or size. Indeed, plant nutrient demand and morphological structure (e.g., root morphology), which vary with ontogeny (Álvarez-Yépiz et al., 2014), may contribute to the discrepancies mentioned above. For example, mature trees featured with larger and deeper root system could help to uptake more available soil P which mainly originates from rock weathering, while available soil N mostly comes from atmospheric deposition in nature ecosystems (Verhoeven and Schmitz, 1991; Güsewell, 2004).

Leaf nutrient concentrations were generally closely correlated with soil available nutrients (Koerselman and Meuleman, 1996; Tessier and Raynal, 2003; Ordoñez et al., 2009; Wang et al., 2017). The mean value of the leaf N:P ratio in the present study was 14.3 ± 1.48 for mature trees and 17.7 ± 1.08 for juveniles for the growing season (**Supplementary Figure S5**), which may indicate that the juveniles are slightly limited by P according to the threshold for P-limitation established by Koerselman and Meuleman (1996), but this case is still not limited by P availability according to the relaxed threshold suggested by Güsewell (2004). Sun et al. (2015) found that the mean N, P concentration and the N:P ratio of Chinese cork oak leaves were 19.00 ± 0.26 mg g^-1^, 1.03 ± 0.03 mg g^-1^, and 20.48 ± 0.63, respectively, across the distribution range of that species in China. Hence, leaf N:P ratio in the present study was lower than that of Sun et al. (2015) but similar to that of Wu et al. (2012) (16.56 for *Q. variabilis*). In line with our results of P concentration (1.13 ± 0.07 mg g^-1^ for juvenile, 1.63 ± 0.18 mg g^-1^ for mature), Han et al. (2005) pointed out that low leaf P across China’s flora, compared to the global average revealed by Reich and Oleksyn (2004) (P: 1.77 mg g^-1^), is a result of low soil P content in China. On the other hand, we found that the N:P ratio in leaves and roots of mature trees increased with latitude (**Supplementary Figure S6**), which is a result of increased tissue N accompanied by relatively stable P concentration across latitudes. A higher leaf N concentration in plants in colder habitats is generally considered as an adaptation mechanism that enhances the metabolic activity and growth rates under low temperatures or short growing season (Reich and Oleksyn, 2004; Soolanayakanahally et al., 2009; El Zein et al., 2011). Fajardo and Piper (2017) also found that nutrient limitation is not likely to be involved in the carbon limitations and could not be an explanation for altitudinal tree-line formation. Our recent field investigation found that the Chinese cork oak showed increasing annual basal increment with increasing latitude from south to north (Gao et al., 2018). We therefore speculate that higher availability of resources (e.g., NSC, N, and P) found in northern populations supports higher growth rate, which in turn decreases the resource storage and remains the resource availability at a stable level.

The fact that more pronounced negative correlations between resource storage and climatic variables (e.g., mean temperature) were found in the dormant season (**Figure 4B**) implies that the projected climate warming could alter trajectories in plant resource allocation into growth and regeneration (De Frenne et al., 2012; Lapenis et al., 2013; Carón et al., 2015), especially as the climate warming in China is predicted to be strongest during the winter months (Ge et al., 2013). This in turn may result in distribution range shift. The results presented here are based on one species with two sampling dates, and should be supplemented by further studies carried out at the community or ecosystem level with multiple species and spanning multiple years, to take into account species-specific responses to environmental variations (Drobyshev et al., 2013; Boisvert-Marsh et al., 2014) as well as their inter-annual fluctuations (Scartazza et al., 2013). Meanwhile, the other factors such as recruitment potential (Graignic et al., 2014), competitive interaction (Ettinger and HilleRisLambers, 2013; Liang et al., 2016), and disturbance (Slaton, 2015; Renwick et al., 2016) had been found to affect distribution range, which should be considered in further exploring the mechanism determining tree species’ northern distribution range other than resource availability.

## Conclusion

In the present study, from the perspective of carbon and nutrient allocation strategy, a whole-tree approach integrating with two distinct life stages was used to explore the mechanisms that determine tree latitudinal range limit, with a case study of Chinese cork oak. Our results indicate that tissue NSCs (soluble sugar, starch and sum of soluble sugar and starch, NSC) and nutrient concentrations (nitrogen and phosphorus) in both mature and juvenile trees did not decrease with increasing latitude across seasons, and even root nitrogen and root NSC in the juvenile trees increased with latitude. Our results suggest that available carbon, N, and P may not be the determinant factors driving the latitudinal range limit of the species studied. These findings will greatly improve our understanding of the mechanism involved in determining the latitudinal range limit, and help to understand and predict the dynamics of the northern range under global warming.

## Author Contributions

J-FL, M-HL, and J-PL conceived the experiment. Y-PD, X-FW, and Y-YN conducted the experiment and analyzed the samples. J-FL, M-HL, QW, W-FX, and Z-PJ wrote the paper. All authors contributed critically to the drafts and gave final approval for publication.

## Conflict of Interest Statement

The authors declare that the research was conducted in the absence of any commercial or financial relationships that could be construed as a potential conflict of interest.

## Footnotes

1 http://data.cma.cn

2 http://globalchange.bnu.edu.cn

3 http://www.rstudio.com/
