# Supplementary material for: The Concentration of Non-structural Carbohydrates, N, and P in Quercus variabilis Does Not Decline Toward Its Northernmost Distribution Range Along a 1500 km Transect in China
Source: Front Plant Sci. 2018 Oct 17;9:1444. doi: 10.3389/fpls.2018.01444 (PMC6199963; doi:10.3389/fpls.2018.01444)

Supporting Information

Table S1 The geographic characteristics of the *Quercus variabilis* sampling sites. The sampling date was estimated using Gong & Jian's (1983) method in terms of biological activity starting from a "standard" date of August 20 and December 13 in Pinggu (Beijing, at 40°N) for growing season and dormant season, respectively.

Sites	Latitude	Longitude	Altitude /m	MAT/℃	MinT/℃	MAP/mm	slope /°	slope direction	canopy density	Approximate Date at same phenological phase	
										growing season	dormant season
Pinggu, Beijing	N40°16′	E117°07′	229~328	10.3	-11.9	590	19~31	S, SW	0.6~0.8	20 Aug	13 Dec
Lincheng, Hebei	N37°28'	E114°05'	646~702	9.9	-9.8	535	15~42	SW	0.6~0.7	14 Aug	19 Dec
Jiyuan, Henan	N35°01′	E112°28′	373~450	12.5	-5.9	642	17~33	SW, SE	0.6~0.7	9 Aug	14 Dec
Neixiang, Henan	N33°29′	E111°54′	861~960	11.4	-4.8	875	19~42	S, SW, SE	0.6~0.8	6 Aug	17 Dec
Zigui, Hubei	N30°46'	E110°20'	874~1034	9.8	-3.4	1620	6~18	SW	0.4~0.5	1 Aug	22 Dec
Cili, Hunan	N29°26'	E110°54'	558~697	14.3	-0.2	1507	5~12	SE, SW	0.6~0.8	26 Jul	28 Dec
Chengbu, Hunan	N26°18′	E110°07′	968~1220	12.7	2.0	1604	20~45	S, SW	0.6~0.7	22 Jul	3Jan

MAT: mean annual temperature; MinT: mean minimum temperature in December; MAP: mean annual precipitation.

Phenological date (PD) was estimated by the equation (Gong & Jian, 1983): $\mathrm{PD} = \mathrm{a} + \mathrm{b}(\mathrm{latitude} - 30°) + \mathrm{c}(\mathrm{longitude} - 110°) + \mathrm{d} * \mathrm{elevation}$; here we use the coefficients (b, c and d) of the species (*Castanea mollissima*) from the same family (Fagaceae), with 2.02 day degree^{-1}, 0.90 day degree^{-1}and 1.00 day per 100 meter, respectively; and the standard date (coefficient a) is set as August 20 for the growing season and December 13 for the dormant season in Pinggu, Beijing.

Fig.S1 Boxplot for concentrations (mg/g) of soluble sugar, starch, non-structural carbohydrates, and soluble sugar: starch ratio. Boxes indicate the lower and upper quartiles. Thick Horizontal lines and red dots represent the median and the mean (both life stages), respectively. For each box, upside or bottom asterisk(s) denote significant difference between the two sampling season or between life stages within a certain season, respectively. *: p<0.05; **: p<0.01; ***: p<0.001.

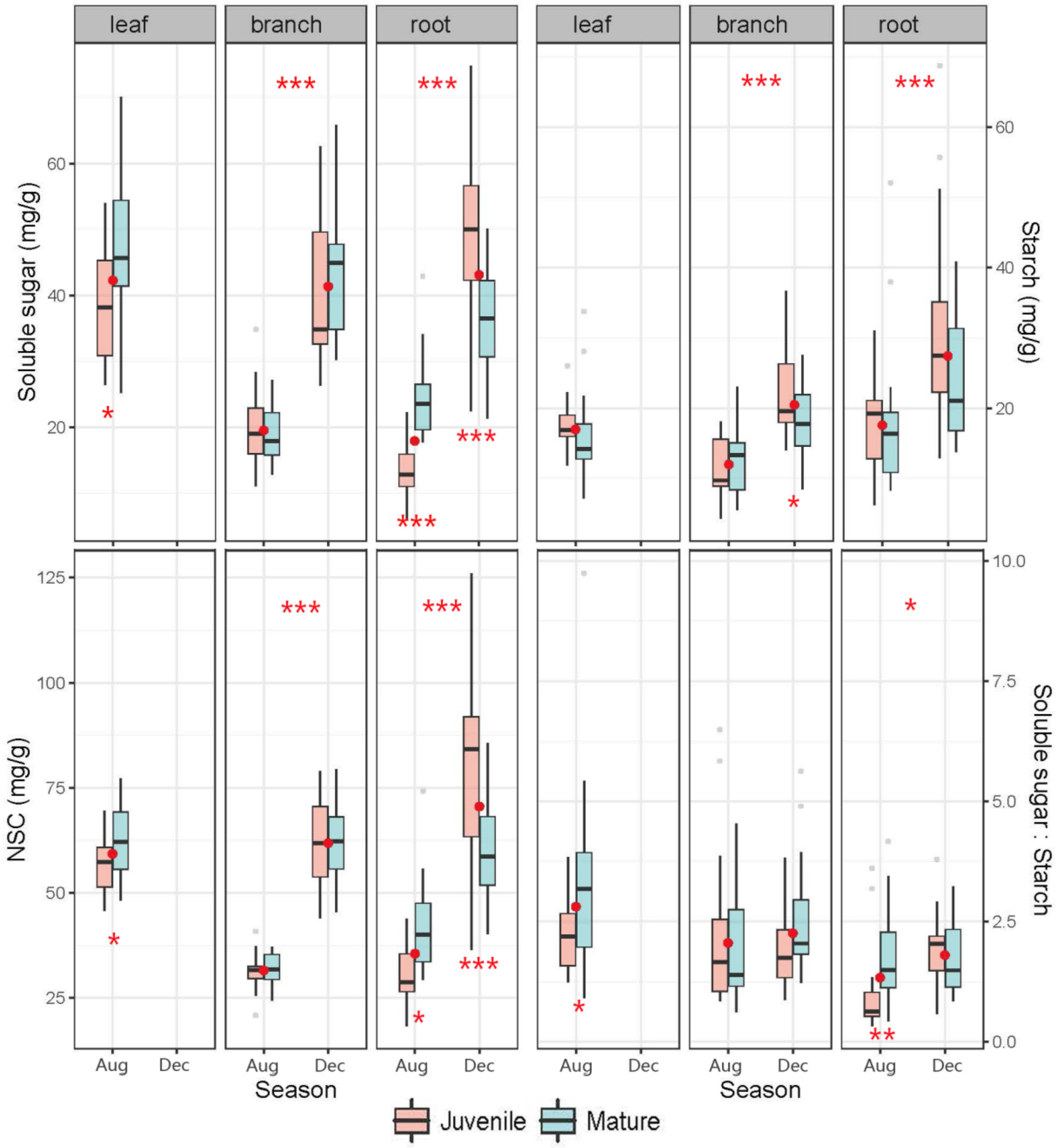

Fig.S2 The soluble sugar concentrations (mg/g) (mean ± se, n=3) across life stages (juveniles vs mature), tissues (leaf, branch and fine root) and sampling seasons (growing season: Aug, 2014; and dormant season: Dec, 2014) along the latitudinal gradient. For each subplot, blue color denotes mature individuals and red for juveniles; fitted curves, determinant coefficients and p values of simple linear regression were given.

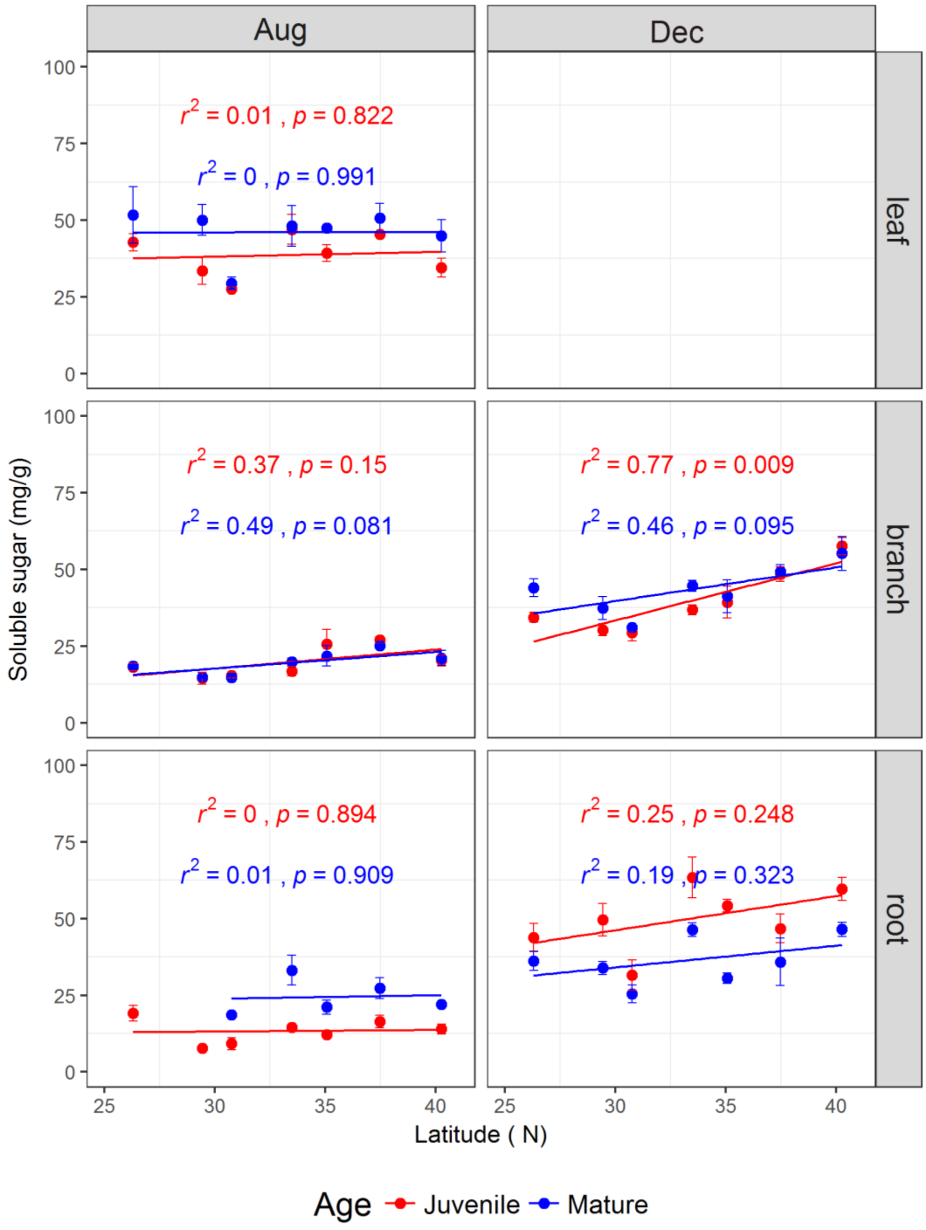

Fig. S3 The starch concentrations (mg/g) (mean ± se, n=3) across life stages (juveniles vs mature), tissues (leaf, branch and fine root) and sampling seasons (growing season: Aug, 2014; and dormant season: Dec, 2014) along the latitudinal gradient. For each subplot, blue color denotes mature individuals and red for juveniles; fitted curves, determinant coefficients and p values of simple linear regression were given.

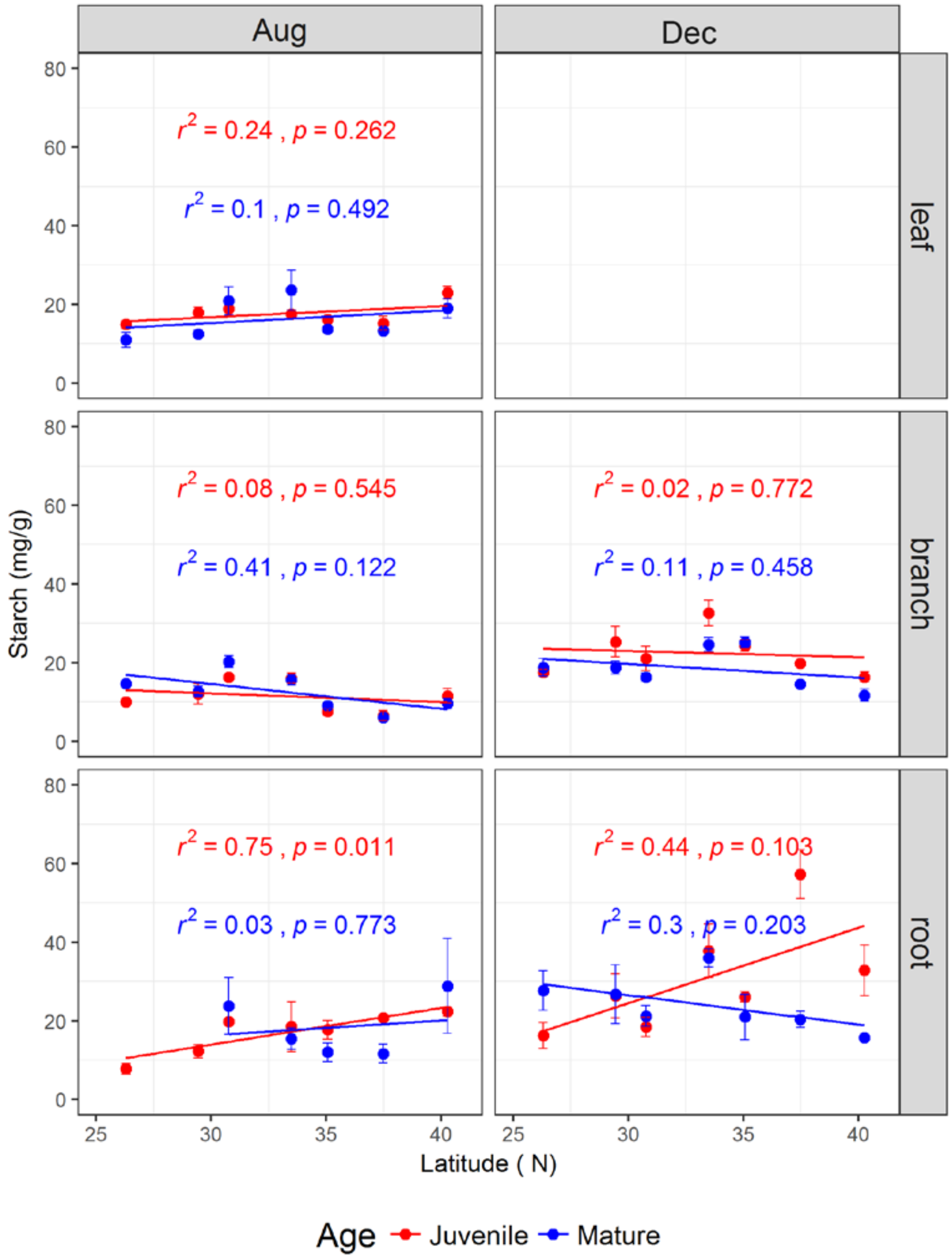

Fig. S4 The ratio of soluble sugar to starch (mean ± se, n=3) across life stages (juveniles vs mature), tissues (leaf, branch and fine root) and sampling seasons (growing season: Aug, 2014; and dormant season: Dec, 2014) along the latitudinal gradient. For each subplot, blue color denotes mature individuals and red for juveniles; fitted curves, determinant coefficients and p values of simple linear regression were given.

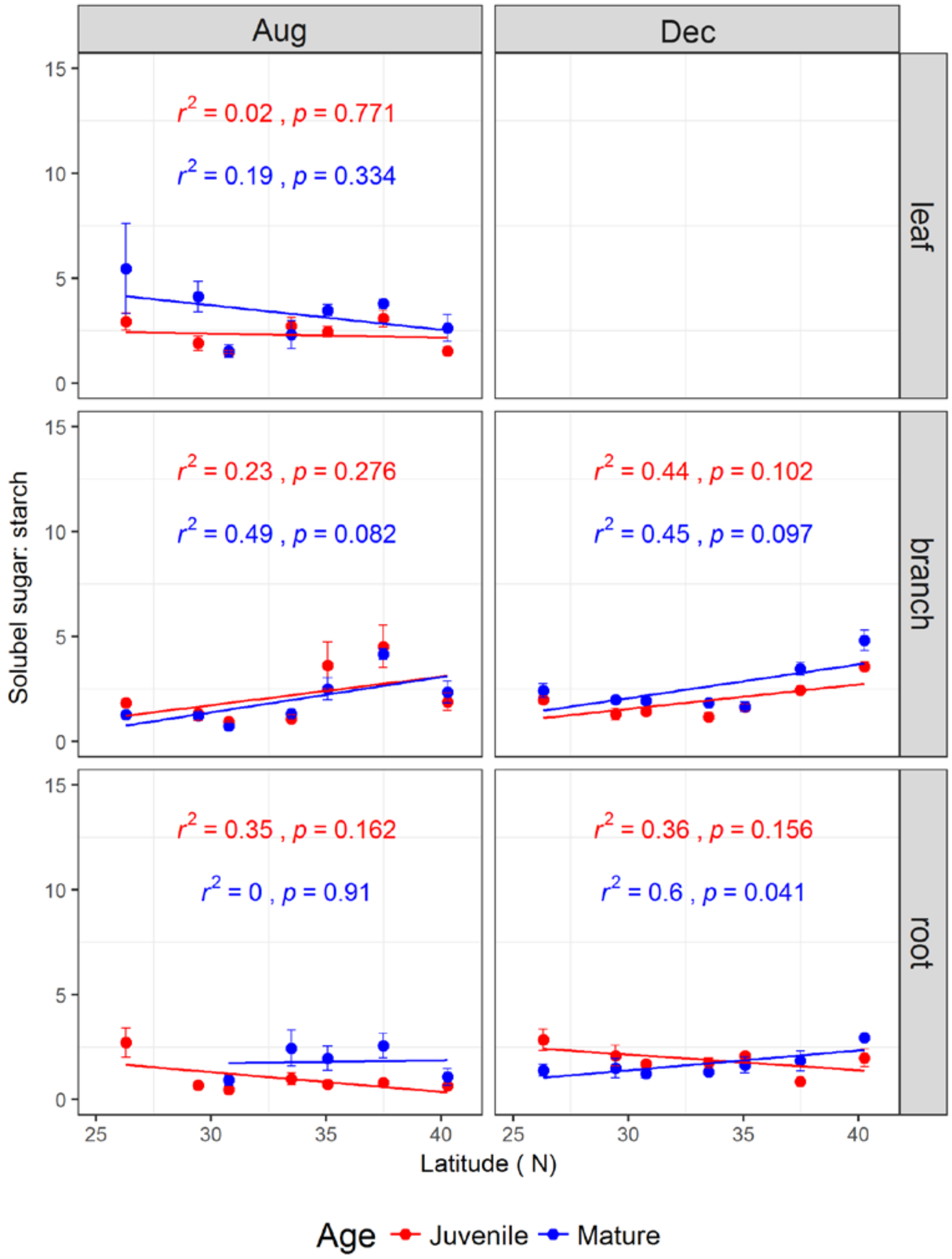

Fig.S5 Boxplot for concentrations (mg/g) of total nitrogen, total phosphorus, and nitrogen: phosphorus ratio. Boxes indicate the lower and upper quartiles. Thick Horizontal lines and red dots represent the median and the mean (both life stages), respectively. For each box, upside or bottom asterisk(s) denote significant difference between the two sampling season or between life stages within a certain season, respectively. *: p<0.05; **: p<0.01; ***: p<0.001.

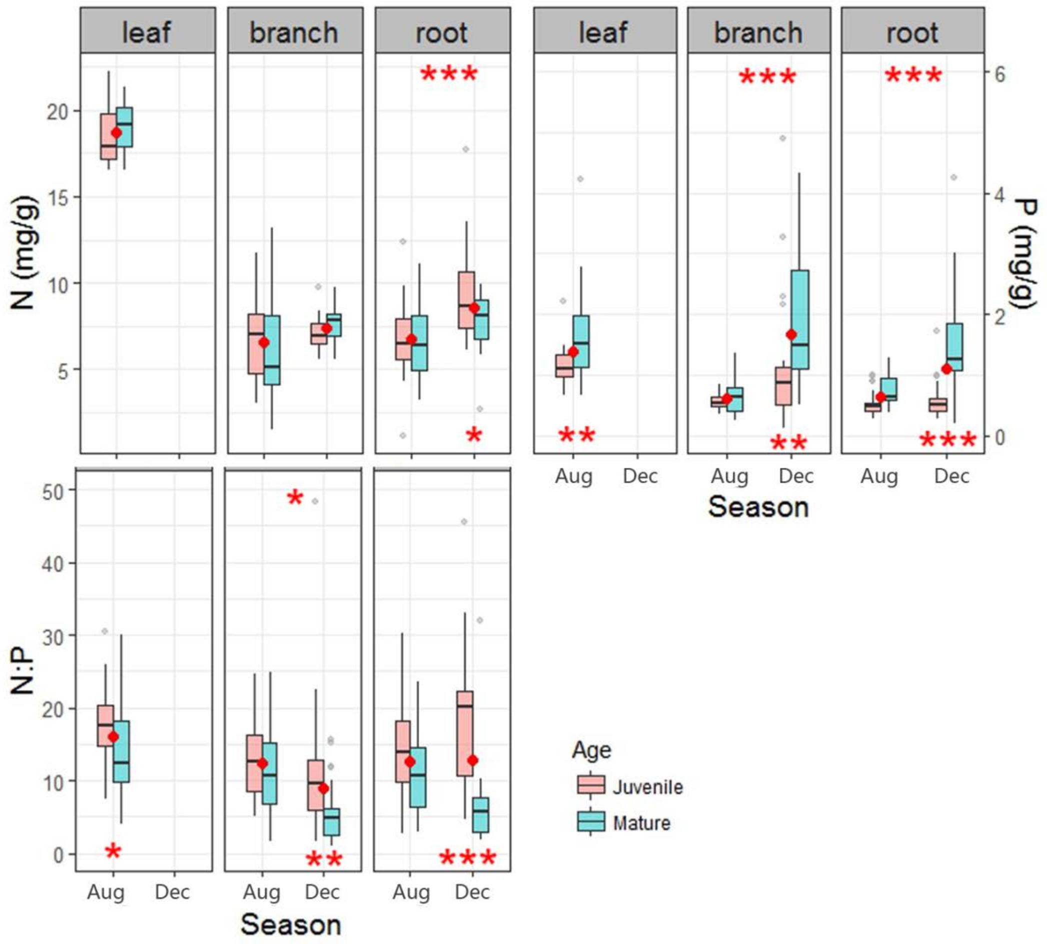

Fig. S6 The ratio of nitrogen to phosphorus (mean ± se, n=3) across life stages (juveniles vs mature), tissues (leaf, branch and fine root) and sampling seasons (growing season: Aug, 2014; and dormant season: Dec, 2014) along the latitudinal gradient. For each subplot, blue color denotes mature individuals and red for juveniles; fitted curves, determinant coefficients and p values of simple linear regression were given.

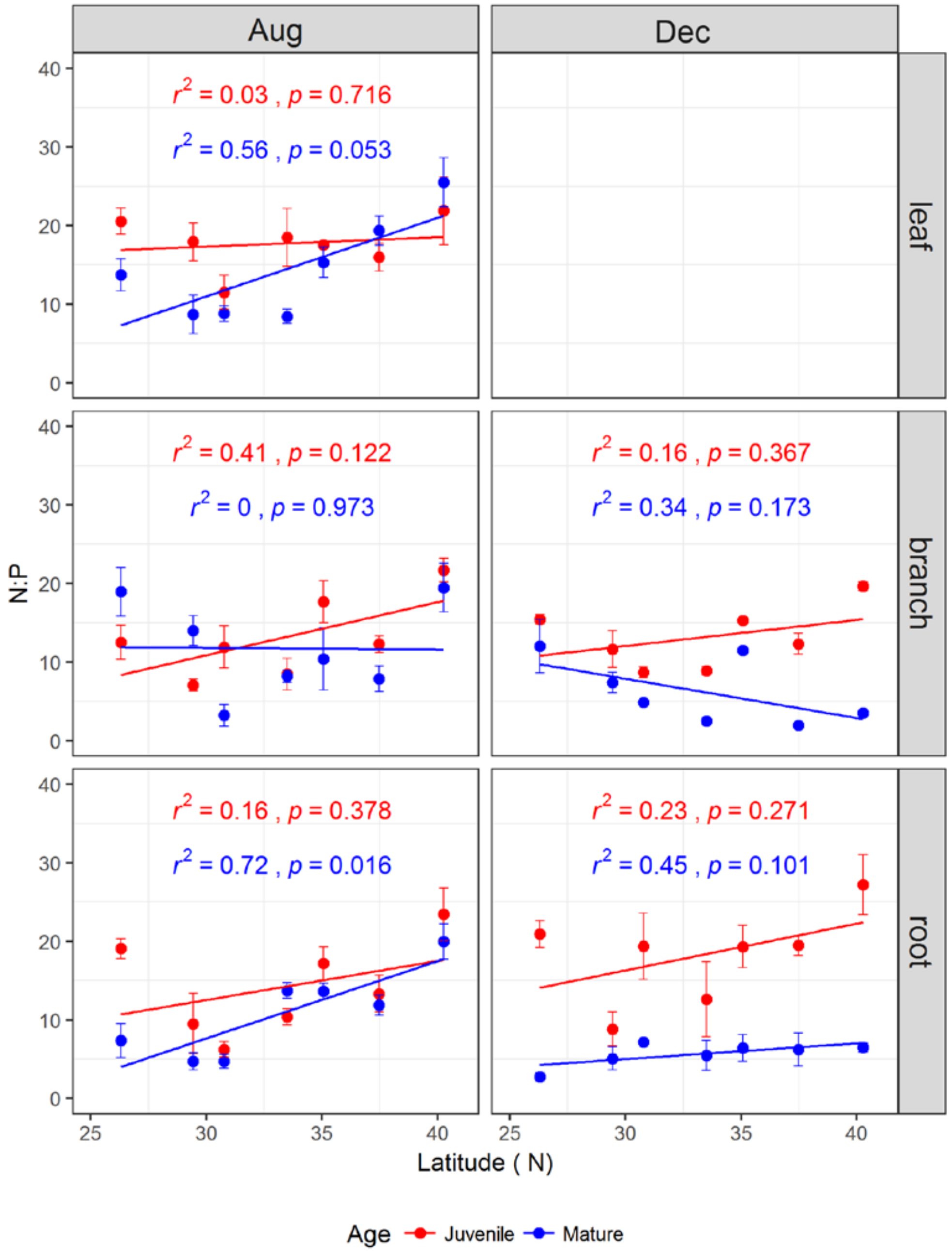

Fig.S7 Variation partitioning (R^2, %) of environment factor and bio-factor in accounting for available resources at two sampling season (A: growing season; B: dormant season). a and b denote the independent effects of environment factor and bio-factor, respectively; ab is their joint effect.

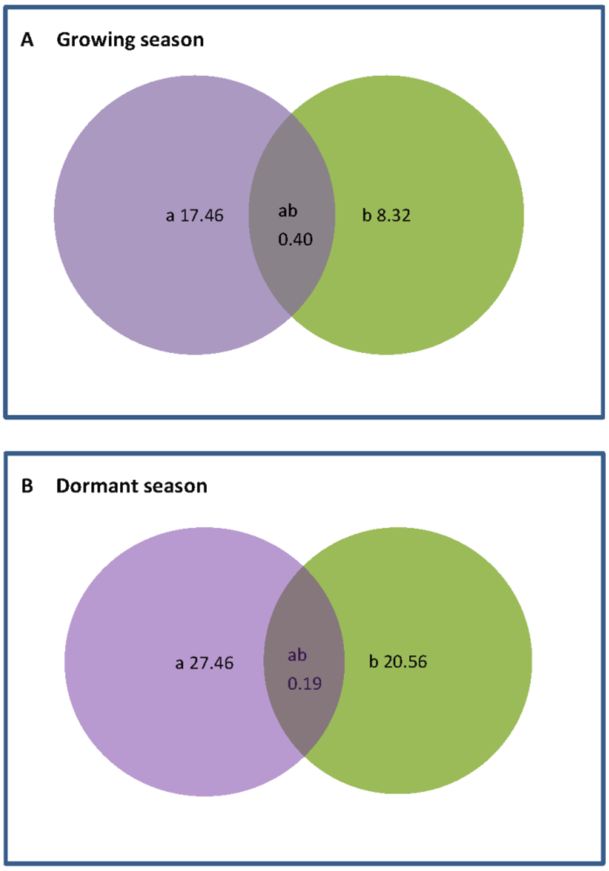

Supplement: Supplementary file 1 [file Image_1.PDF]
